# Crystal structure of 6,6′-dimethyl-2*H*,2′*H*-3,4′-bichromene-2,2′-dione

**DOI:** 10.1107/S1600536814021825

**Published:** 2014-10-11

**Authors:** Kiran K. Pujar, Manohar V. Kulkarni, G. N. Anil Kumar

**Affiliations:** a Department of Chemistry, Karnatak University, Dharwad 580 010, India; b Department of Physics, M S Ramaiah Institute of Technology, MSRIT Post, Bangalore 560 054, India

**Keywords:** crystal structure, bicoumarin, hydrogen bonds, π–π inter­actions

## Abstract

In the title compound, C_20_H_14_O_4_, the dihedral angle between the two coumarin ring systems is 52.37 (19)°, showing a *gauche* arrangement across the C—C bond which links the two units. The carbonyl groups of the two coumarin units adopt an s-*trans* arrangement. In the crystal, pairs of C—H⋯O hydrogen bonds and π–π inter­actions [centroid–centroid distance = 3.631 (2) Å] connect the mol­ecules into inversion dimers.

## Abstract 2

In the title compound, the dihedral angle between the two coumarin units is 52.37 (19)°, showing a *gauche* arrangement across the C—C bond which links the two ring systems. In the crystal, C—H⋯O hydrogen bonds connect centrosymmetrically-related mol­ecules into dimers.

## Chemical context   

Bicoumarins, in which two coumarin ring systems are directly linked through a C—C bond, are a group of regio-isomers which are of synthetic inter­est (Ilyas & Parveen, 1996 ▶; Dubovik *et al.*, 2004 ▶; Frasinyuk *et al.*, 2012 ▶). Their natural occurrence and structural diversity originate from various positions of the linkage which can lead to pyran–pyran-linked bicoumarins, *viz*., 3-3′, 3-4′, 4-4′, or pyran–benzene-linked bicoumarins wherein the points of linkage are C3/C4 with the C5–C8 positions in the second coumarin moiety (Hussain *et al.*, 2012 ▶). 3-3′ Bicoumarins isolated from Chinese medicinal plants and Mediterranean sponges (Panichayupakaranant *et al.*, 1998 ▶) have been shown to exhibit insecticidal and anti-proliferative properties. 8-8′ Bicoumarins have shown anti­leukemic, nematocidal and cardiotoxic activity as well as anti­schistosomial, sedative and hypotensive effects (Ulubelen *et al.*, 1986 ▶). 6-8′ Bicoumarins have been evaluated for urease inhibitory activity (Ayaz *et al.*, 2006 ▶). Atropisomerism has been observed for naturally occurring 3-6′ bicoumarins (Zhan *et al.*, 2003 ▶). 5-5′ Bicoumarins competitively inhibit epoxide reductase of vitamin K, preventing the reduction of vitamin K into hydro­quinone, leading to their anti­coagulant activity (Zhou *et al.*, 2009 ▶). 3-8′ Bicoumarins exhibit cytotoxicity towards human solid tumour cell lines, affording ED_50_ values of 7.5, 55, 5.8 µg/ml against non-small-cell-lung carcinoma A-549, breast adenocarcinoma MCF-7, and colon adeno-carcinoma HT-29 cells respectively (Tepaske & Gloer, 1992 ▶).
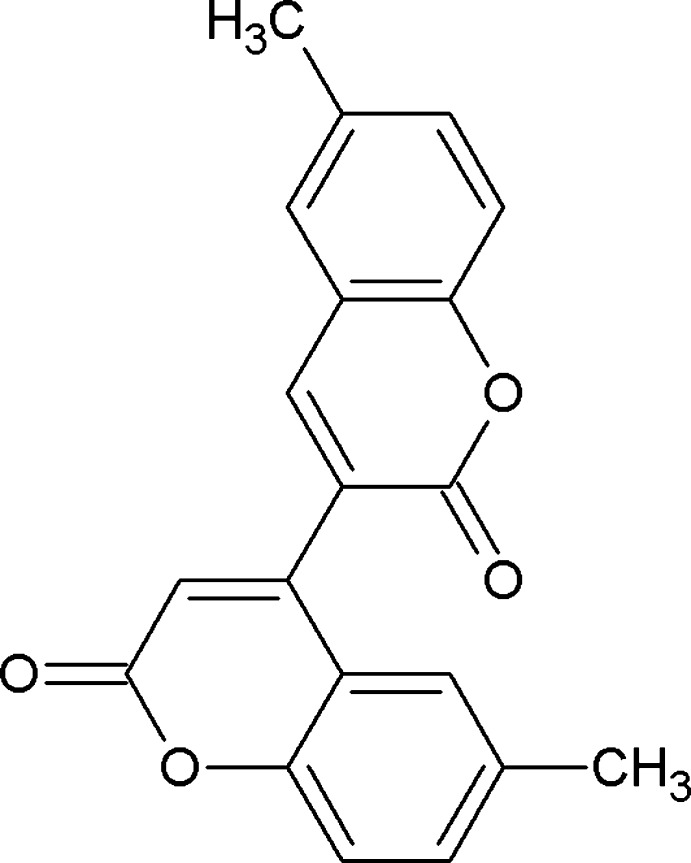


 In view of the above cited activities of directly linked coumarin dimers, the present work reports the synthesis under metal-free conditions of a new 4-3′ bicoumarin and its structure.

## Structural commentary   

**Figure 1:**
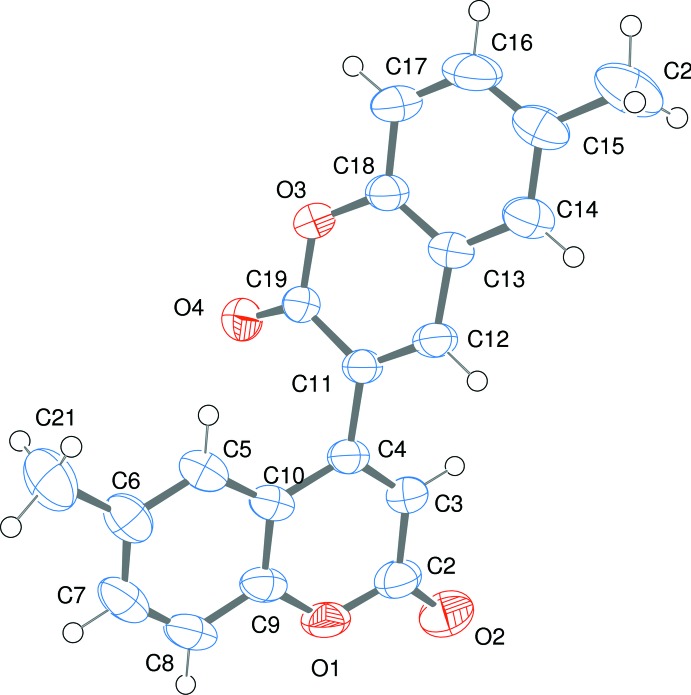
The mol­ecular structure of the title compound, showing the atom-labelling scheme and with displacement ellipsoids drawn at the 50% probability level.

**Figure 2:**
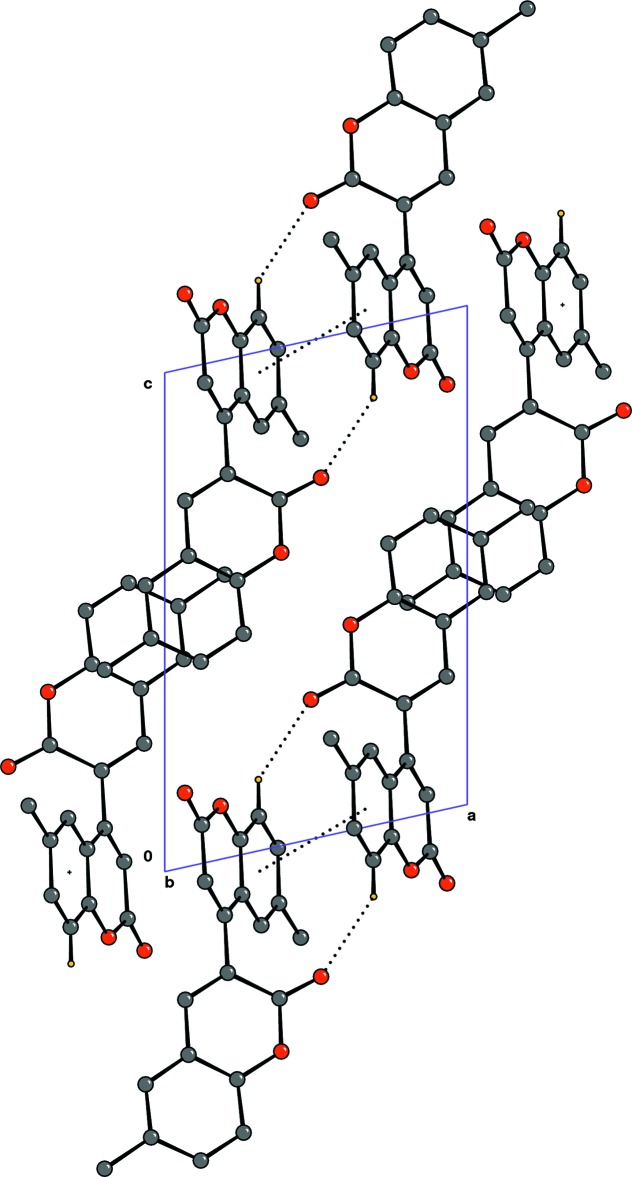
A packing diagram of the title compound, viewed along the *b* axis. Dashed lines indicate C—H⋯O hydrogen bonds and π–π inter­actions. H atoms not involved in hydrogen bonding have been omitted for clarity.

The mol­ecular structure of the title compound is shown in Fig. 1 ▶. The packing viewed along the *b* axis (Fig. 2 ▶) shows the existence of inter­molecular C—H⋯O hydrogen bonds between the carbonyl O4 of one coumarin moiety and the aromatic H8 of the second unit (Table 1 ▶), which has also been observed in a 3-5′ bicoumarin (Fun *et al.*, 2009 ▶). The two coumarin rings exhibit an *s*-*trans* arrangement across the C4—C11 bond for the two double bonds *viz*. C3=C4 and C11=C12. The non-planar nature of the bi-heterocyclic system is revealed through the torsion angles C3—C4—C11—C12 [−52.37 (19)°] and C10—C4—C11—C19 [−59.32 (17)°], which almost corresponds to a *gauche* conformation.

## Supra­molecular features   

In the crystal, pairs of C—H⋯O hydrogen bonds and π–π inter­actions [*Cg*1⋯*Cg*1^i^ = 3.631 (2); slippage = 1.491 Å; *Cg*1 is the centroid of the C5–C10 ring; symmetry code: (i) 1 − *x*, −*y*, −*z*] connect mol­ecules into inversion dimers (Fig. 2 ▶).

## Database survey   

A search of the Cambridge Structural Database (Version 5.35, updates Feb 2014; Groom & Allen, 2014 ▶) revealed two related structures, *viz.* 7,7′,8,8′-tetra­meth­oxy-4,4′-dimethyl-3,5′-bichromene-2,2′-dione (Fun *et al.*, 2009 ▶) and 7,7′-dihy­droxy-4,4′-dimethyl-3,4-di­hydro-2*H*,2′*H*-4,6′-bichromene-2,2′-dione (Pereira Silva *et al.*, 2011 ▶). In these two compounds, the dihedral angles between the coumarin ring systems are 79.93 (3) and 88.07 (2)°, respectively. The corresponding angle in the title compound is 52.37 (19)°.

## Synthesis and Crystallization   

6-Methyl­coumarin 4-acetic acid (0.01 mol) and 5-methyl­salicyl­aldehyde (0.01 mol) were taken in a round-bottomed flask containing (1.5 eq) NaH and 3 ml of acetic anhydride. The flask, fitted with a guard tube, was stirred for 1.5 h. The progress of the reaction was monitored by TLC, the solid that separated was filtered off and washed with diethyl ether and again with 5% NaHCO_3_ to remove unreacted 6-methyl­coumarin 4-acetic acid. Then the solid was dried and recrystallized from ethanol. Crystals suitable for diffraction studies were obtained through slow evaporation from a DMF solution.

## Refinement   

Crystal data, data collection and structure refinement details are summarized in Table 2 ▶. C-bound H atoms were positioned geometrically and allowed to ride on their parent atoms, with C—H = 0.93–0.98 Å and *U*_iso_(H) = 1.2*U*_eq_(C) or 1.5*U*_eq_(C_meth­yl_).

## Supplementary Material

Crystal structure: contains datablock(s) global, I. DOI: 10.1107/S1600536814021825/is5375sup1.cif

Structure factors: contains datablock(s) I. DOI: 10.1107/S1600536814021825/is5375Isup2.hkl

Supporting information file. DOI: 10.1107/S1600536814021825/is5375Isup3.cml

CCDC reference: 1027466

Additional supporting information:  crystallographic information; 3D view; checkCIF report

## Figures and Tables

**Table 1:** Hydrogen-bond geometry (, )

*D*H*A*	*D*H	H*A*	*D* *A*	*D*H*A*
C8H8O4^i^	0.93	2.53	3.330(2)	145

Symmetry code: (i) 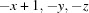.

**Table 2:** Experimental details

Crystal data	
Chemical formula	C_20_H_14_O_4_
*M*_r_	318.31
Crystal system, space group	Triclinic, *P* 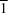
Temperature (K)	296
*a*, *b*, *c* ()	7.834(1), 8.0455(9), 12.7952(15)
, , ()	79.492(5), 77.096(4), 86.637(5)
*V* (^3^)	772.78(16)
*Z*	2
Radiation type	Mo *K*
(mm^1^)	0.10
Crystal size (mm)	0.35 0.31 0.25

Data collection	
Diffractometer	Bruker *SMART* CCD area-detector
Absorption correction	Multi-scan (*SADABS*; Sheldrick, 1996 ▶)
*T*_min_, *T*_max_	0.954, 0.964
No. of measured, independent and observed [*I* > 2(*I*)] reflections	12862, 3499, 2509
*R*_int_	0.027
(sin /)_max_ (^1^)	0.648

Refinement	
*R*[*F* ^2^ > 2(*F* ^2^)], *wR*(*F* ^2^), *S*	0.046, 0.140, 1.05
No. of reflections	3499
No. of parameters	219
H-atom treatment	H-atom parameters constrained
_max_, _min_ (e ^3^)	0.19, 0.20

Computer programs: *SMART* and *SAINT* (Bruker, 1998 ▶), *SIR92* (Altomare *et al.*, 1993 ▶), *SHELXL97* (Sheldrick, 2008 ▶), *ORTEP-3 for Windows* (Farrugia, 2012 ▶), *CAMERON* (Watkin *et al.*, 1996 ▶), *PARST* (Nardelli, 1996 ▶) and *PLATON* (Spek, 2009 ▶).

## supplementary crystallographic information

### Crystal data

**Table:**

C_20_H_14_O_4_	*Z* = 2
*M**_r_* = 318.31	*F*(000) = 332
Triclinic, *P*1	*D*_x_ = 1.368 Mg m^−^^3^
Hall symbol: -P 1	Mo *K*α radiation, λ = 0.71073 Å
*a* = 7.834 (1) Å	Cell parameters from 560 reflections
*b* = 8.0455 (9) Å	θ = 1.7°
*c* = 12.7952 (15) Å	µ = 0.10 mm^−^^1^
α = 79.492 (5)°	*T* = 296 K
β = 77.096 (4)°	Block, white
γ = 86.637 (5)°	0.35 × 0.31 × 0.25 mm
*V* = 772.78 (16) Å^3^	

### Data collection

**Table:**

Bruker SMART CCD area-detector diffractometer	3499 independent reflections
Radiation source: fine-focus sealed tube	2509 reflections with *I* > 2σ(*I*)
Graphite monochromator	*R*_int_ = 0.027
φ and ω scans	θ_max_ = 27.4°, θ_min_ = 1.7°
Absorption correction: multi-scan (*SADABS*; Sheldrick, 1996)	*h* = −10→10
*T*_min_ = 0.954, *T*_max_ = 0.964	*k* = −10→9
12862 measured reflections	*l* = −16→16

### Refinement

**Table:**

Refinement on *F*^2^	Primary atom site location: structure-invariant direct methods
Least-squares matrix: full	Secondary atom site location: difference Fourier map
*R*[*F*^2^ > 2σ(*F*^2^)] = 0.046	Hydrogen site location: inferred from neighbouring sites
*wR*(*F*^2^) = 0.140	H-atom parameters constrained
*S* = 1.05	*w* = 1/[σ^2^(*F*_o_^2^) + (0.0661*P*)^2^ + 0.1433*P*] where *P* = (*F*_o_^2^ + 2*F*_c_^2^)/3
3499 reflections	(Δ/σ)_max_ < 0.001
219 parameters	Δρ_max_ = 0.19 e Å^−^^3^
0 restraints	Δρ_min_ = −0.20 e Å^−^^3^

### Special details

**Table:**

Geometry. All e.s.d.'s (except the e.s.d. in the dihedral angle between two l.s. planes) are estimated using the full covariance matrix. The cell e.s.d.'s are taken into account individually in the estimation of e.s.d.'s in distances, angles and torsion angles; correlations between e.s.d.'s in cell parameters are only used when they are defined by crystal symmetry. An approximate (isotropic) treatment of cell e.s.d.'s is used for estimating e.s.d.'s involving l.s. planes.
Refinement. Refinement of *F*^2^ against ALL reflections. The weighted *R*-factor *wR* and goodness of fit *S* are based on *F*^2^, conventional *R*-factors *R* are based on *F*, with *F* set to zero for negative *F*^2^. The threshold expression of *F*^2^ > σ(*F*^2^) is used only for calculating *R*-factors(gt) *etc*. and is not relevant to the choice of reflections for refinement. *R*-factors based on *F*^2^ are statistically about twice as large as those based on *F*, and *R*- factors based on ALL data will be even larger.

### Fractional atomic coordinates and isotropic or equivalent isotropic displacement parameters (Å^2^)

**Table:**

	*x*	*y*	*z*	*U*_iso_*/*U*_eq_	
O1	0.81516 (14)	0.16314 (15)	−0.10679 (8)	0.0577 (3)	
O2	0.9332 (2)	0.4150 (2)	−0.14906 (10)	0.0853 (4)	
O3	0.61479 (14)	0.15867 (15)	0.41223 (8)	0.0569 (3)	
O4	0.48324 (15)	0.13864 (15)	0.27995 (9)	0.0603 (3)	
C2	0.8773 (2)	0.2996 (2)	−0.07754 (13)	0.0583 (4)	
C3	0.8662 (2)	0.2942 (2)	0.03783 (11)	0.0508 (4)	
H3	0.9052	0.3863	0.06	0.061*	
C4	0.80175 (18)	0.16160 (17)	0.11386 (11)	0.0405 (3)	
C5	0.6808 (2)	−0.13246 (17)	0.15031 (12)	0.0478 (4)	
H5	0.6783	−0.1418	0.2242	0.057*	
C6	0.6220 (2)	−0.26560 (18)	0.11392 (14)	0.0547 (4)	
C7	0.6262 (2)	−0.2483 (2)	0.00295 (15)	0.0595 (5)	
H7	0.5852	−0.3356	−0.0229	0.071*	
C8	0.6891 (2)	−0.1057 (2)	−0.06922 (13)	0.0560 (4)	
H8	0.6905	−0.0967	−0.1429	0.067*	
C9	0.75025 (18)	0.0239 (2)	−0.03095 (12)	0.0460 (4)	
C10	0.74384 (18)	0.01530 (17)	0.08010 (11)	0.0414 (3)	
C11	0.79180 (18)	0.16893 (16)	0.23068 (10)	0.0386 (3)	
C12	0.93182 (19)	0.20659 (19)	0.26592 (11)	0.0450 (3)	
H12	1.0386	0.2245	0.2165	0.054*	
C13	0.92131 (19)	0.21999 (17)	0.37755 (11)	0.0433 (3)	
C14	1.0631 (2)	0.2583 (2)	0.41766 (13)	0.0540 (4)	
H14	1.1719	0.277	0.3705	0.065*	
C15	1.0449 (2)	0.2688 (2)	0.52638 (13)	0.0572 (4)	
C16	0.8808 (3)	0.2377 (2)	0.59489 (13)	0.0679 (5)	
H16	0.8669	0.2432	0.6683	0.081*	
C17	0.7393 (3)	0.1995 (2)	0.55805 (13)	0.0680 (5)	
H17	0.6312	0.1788	0.6056	0.082*	
C18	0.7602 (2)	0.19221 (18)	0.44840 (11)	0.0472 (4)	
C19	0.6205 (2)	0.15326 (17)	0.30488 (11)	0.0440 (3)	
C20	1.1984 (3)	0.3134 (3)	0.56865 (17)	0.0830 (6)	
H20A	1.2798	0.2195	0.5708	0.124*	
H20B	1.1575	0.3386	0.6407	0.124*	
H20C	1.2554	0.4103	0.5214	0.124*	
C21	0.5504 (3)	−0.4212 (2)	0.19269 (18)	0.0784 (6)	
H21A	0.6207	−0.4505	0.2462	0.118*	
H21B	0.5529	−0.5131	0.1539	0.118*	
H21C	0.4319	−0.3991	0.2282	0.118*	

### Atomic displacement parameters (Å^2^)

**Table:**

	*U*^11^	*U*^22^	*U*^33^	*U*^12^	*U*^13^	*U*^23^
O1	0.0565 (7)	0.0856 (8)	0.0318 (5)	−0.0030 (6)	−0.0070 (5)	−0.0147 (5)
O2	0.1050 (11)	0.1025 (10)	0.0394 (6)	−0.0284 (8)	−0.0057 (7)	0.0071 (7)
O3	0.0581 (7)	0.0744 (7)	0.0360 (5)	−0.0229 (5)	0.0050 (5)	−0.0154 (5)
O4	0.0469 (7)	0.0795 (8)	0.0575 (7)	−0.0158 (6)	−0.0036 (5)	−0.0243 (6)
C2	0.0556 (10)	0.0799 (11)	0.0365 (8)	−0.0071 (8)	−0.0048 (7)	−0.0074 (8)
C3	0.0521 (9)	0.0634 (9)	0.0358 (7)	−0.0107 (7)	−0.0039 (7)	−0.0101 (7)
C4	0.0378 (7)	0.0510 (8)	0.0335 (7)	0.0029 (6)	−0.0065 (6)	−0.0118 (6)
C5	0.0586 (9)	0.0446 (7)	0.0461 (8)	0.0105 (6)	−0.0207 (7)	−0.0154 (6)
C6	0.0581 (10)	0.0444 (8)	0.0694 (10)	0.0120 (7)	−0.0236 (8)	−0.0224 (7)
C7	0.0564 (10)	0.0631 (10)	0.0734 (11)	0.0133 (8)	−0.0241 (9)	−0.0415 (9)
C8	0.0469 (9)	0.0817 (11)	0.0496 (9)	0.0154 (8)	−0.0155 (7)	−0.0367 (8)
C9	0.0380 (8)	0.0641 (9)	0.0394 (7)	0.0111 (6)	−0.0098 (6)	−0.0204 (7)
C10	0.0409 (8)	0.0496 (8)	0.0377 (7)	0.0100 (6)	−0.0129 (6)	−0.0162 (6)
C11	0.0453 (8)	0.0393 (6)	0.0306 (6)	−0.0017 (5)	−0.0050 (6)	−0.0079 (5)
C12	0.0421 (8)	0.0590 (8)	0.0336 (7)	0.0011 (6)	−0.0030 (6)	−0.0146 (6)
C13	0.0521 (9)	0.0452 (7)	0.0338 (7)	0.0034 (6)	−0.0103 (6)	−0.0105 (6)
C14	0.0546 (10)	0.0674 (10)	0.0456 (8)	0.0091 (7)	−0.0174 (7)	−0.0195 (7)
C15	0.0792 (12)	0.0534 (9)	0.0477 (9)	0.0131 (8)	−0.0316 (9)	−0.0139 (7)
C16	0.1023 (15)	0.0711 (11)	0.0322 (8)	−0.0038 (10)	−0.0178 (9)	−0.0092 (7)
C17	0.0863 (13)	0.0816 (12)	0.0322 (8)	−0.0220 (10)	0.0009 (8)	−0.0106 (8)
C18	0.0623 (10)	0.0451 (7)	0.0328 (7)	−0.0093 (7)	−0.0048 (7)	−0.0074 (6)
C19	0.0517 (9)	0.0426 (7)	0.0367 (7)	−0.0106 (6)	−0.0025 (6)	−0.0101 (6)
C20	0.0974 (15)	0.1004 (15)	0.0710 (12)	0.0216 (12)	−0.0535 (12)	−0.0306 (11)
C21	0.1041 (16)	0.0426 (8)	0.0973 (15)	0.0001 (9)	−0.0352 (12)	−0.0189 (9)

### Geometric parameters (Å, º)

**Table:**

O1—C2	1.370 (2)	C11—C12	1.345 (2)
O1—C9	1.3805 (19)	C11—C19	1.458 (2)
O2—C2	1.204 (2)	C12—C13	1.4358 (18)
O3—C19	1.3728 (17)	C12—H12	0.93
O3—C18	1.3793 (18)	C13—C18	1.383 (2)
O4—C19	1.2055 (18)	C13—C14	1.394 (2)
C2—C3	1.452 (2)	C14—C15	1.384 (2)
C3—C4	1.344 (2)	C14—H14	0.93
C3—H3	0.93	C15—C16	1.392 (3)
C4—C10	1.4538 (18)	C15—C20	1.509 (2)
C4—C11	1.4911 (17)	C16—C17	1.368 (3)
C5—C6	1.383 (2)	C16—H16	0.93
C5—C10	1.395 (2)	C17—C18	1.387 (2)
C5—H5	0.93	C17—H17	0.93
C6—C7	1.394 (2)	C20—H20A	0.96
C6—C21	1.506 (2)	C20—H20B	0.96
C7—C8	1.374 (2)	C20—H20C	0.96
C7—H7	0.93	C21—H21A	0.96
C8—C9	1.381 (2)	C21—H21B	0.96
C8—H8	0.93	C21—H21C	0.96
C9—C10	1.3997 (18)		
			
C2—O1—C9	121.88 (12)	C13—C12—H12	119
C19—O3—C18	122.41 (11)	C18—C13—C14	118.92 (13)
O2—C2—O1	117.52 (15)	C18—C13—C12	117.22 (13)
O2—C2—C3	125.51 (17)	C14—C13—C12	123.86 (14)
O1—C2—C3	116.95 (14)	C15—C14—C13	121.18 (16)
C4—C3—C2	122.65 (14)	C15—C14—H14	119.4
C4—C3—H3	118.7	C13—C14—H14	119.4
C2—C3—H3	118.7	C14—C15—C16	117.86 (15)
C3—C4—C10	119.22 (12)	C14—C15—C20	120.80 (17)
C3—C4—C11	118.88 (12)	C16—C15—C20	121.34 (16)
C10—C4—C11	121.90 (12)	C17—C16—C15	122.34 (15)
C6—C5—C10	122.28 (14)	C17—C16—H16	118.8
C6—C5—H5	118.9	C15—C16—H16	118.8
C10—C5—H5	118.9	C16—C17—C18	118.71 (16)
C5—C6—C7	117.80 (15)	C16—C17—H17	120.6
C5—C6—C21	120.70 (15)	C18—C17—H17	120.6
C7—C6—C21	121.46 (15)	O3—C18—C13	121.18 (12)
C8—C7—C6	121.81 (14)	O3—C18—C17	117.84 (14)
C8—C7—H7	119.1	C13—C18—C17	120.98 (15)
C6—C7—H7	119.1	O4—C19—O3	117.19 (13)
C7—C8—C9	119.16 (14)	O4—C19—C11	125.75 (13)
C7—C8—H8	120.4	O3—C19—C11	117.05 (13)
C9—C8—H8	120.4	C15—C20—H20A	109.5
C8—C9—O1	117.05 (13)	C15—C20—H20B	109.5
C8—C9—C10	121.36 (15)	H20A—C20—H20B	109.5
O1—C9—C10	121.58 (13)	C15—C20—H20C	109.5
C5—C10—C9	117.54 (13)	H20A—C20—H20C	109.5
C5—C10—C4	124.84 (12)	H20B—C20—H20C	109.5
C9—C10—C4	117.62 (13)	C6—C21—H21A	109.5
C12—C11—C19	119.82 (12)	C6—C21—H21B	109.5
C12—C11—C4	121.73 (12)	H21A—C21—H21B	109.5
C19—C11—C4	118.09 (12)	C6—C21—H21C	109.5
C11—C12—C13	121.94 (13)	H21A—C21—H21C	109.5
C11—C12—H12	119	H21B—C21—H21C	109.5
			
C9—O1—C2—O2	179.27 (14)	C3—C4—C11—C19	120.68 (15)
C9—O1—C2—C3	−2.2 (2)	C10—C4—C11—C19	−59.32 (17)
O2—C2—C3—C4	179.75 (17)	C19—C11—C12—C13	4.7 (2)
O1—C2—C3—C4	1.3 (2)	C4—C11—C12—C13	177.64 (12)
C2—C3—C4—C10	1.3 (2)	C11—C12—C13—C18	0.1 (2)
C2—C3—C4—C11	−178.68 (14)	C11—C12—C13—C14	179.81 (13)
C10—C5—C6—C7	−0.4 (2)	C18—C13—C14—C15	−0.1 (2)
C10—C5—C6—C21	−178.33 (15)	C12—C13—C14—C15	−179.77 (14)
C5—C6—C7—C8	1.2 (2)	C13—C14—C15—C16	0.9 (2)
C21—C6—C7—C8	179.09 (15)	C13—C14—C15—C20	−178.91 (15)
C6—C7—C8—C9	0.1 (2)	C14—C15—C16—C17	−0.7 (3)
C7—C8—C9—O1	178.84 (13)	C20—C15—C16—C17	179.08 (17)
C7—C8—C9—C10	−2.2 (2)	C15—C16—C17—C18	−0.3 (3)
C2—O1—C9—C8	179.28 (14)	C19—O3—C18—C13	−0.9 (2)
C2—O1—C9—C10	0.3 (2)	C19—O3—C18—C17	178.21 (14)
C6—C5—C10—C9	−1.6 (2)	C14—C13—C18—O3	178.15 (13)
C6—C5—C10—C4	177.80 (13)	C12—C13—C18—O3	−2.1 (2)
C8—C9—C10—C5	2.9 (2)	C14—C13—C18—C17	−1.0 (2)
O1—C9—C10—C5	−178.19 (12)	C12—C13—C18—C17	178.77 (14)
C8—C9—C10—C4	−176.54 (13)	C16—C17—C18—O3	−178.01 (15)
O1—C9—C10—C4	2.4 (2)	C16—C17—C18—C13	1.1 (3)
C3—C4—C10—C5	177.47 (13)	C18—O3—C19—O4	−173.26 (13)
C11—C4—C10—C5	−2.5 (2)	C18—O3—C19—C11	5.6 (2)
C3—C4—C10—C9	−3.1 (2)	C12—C11—C19—O4	171.31 (14)
C11—C4—C10—C9	176.86 (12)	C4—C11—C19—O4	−1.9 (2)
C3—C4—C11—C12	−52.37 (19)	C12—C11—C19—O3	−7.45 (19)
C10—C4—C11—C12	127.63 (15)	C4—C11—C19—O3	179.36 (11)

### Hydrogen-bond geometry (Å, º)

**Table:**

*D*—H···*A*	*D*—H	H···*A*	*D*···*A*	*D*—H···*A*
C8—H8···O4^i^	0.93	2.53	3.330 (2)	145

Symmetry code: (i) −*x*+1, −*y*, −*z*.

## References

[bb1] Altomare, A., Cascarano, G., Giacovazzo, C. & Guagliardi, A. (1993). *J. Appl. Cryst.* **26**, 343–350.

[bb2] Ayaz, M., Riaz, M. A. M., Haq, A. U., Malik, A. & Choudhary, M. I. (2006). *J. Enzyme Inhib. Med. Chem.* **49**, 527–529. 10.1080/14756360600774470 17194022

[bb3] Bruker (1998). *SMART* and *SAINT*. Bruker AXS Inc., Madison, Wisconsin, USA.

[bb4] Dubovik, I. P., Garazd, M. M. & Khilya, V. P. (2004). *Chem. Nat. Compd*, **40**, 434–443.

[bb5] Farrugia, L. J. (2012). *J. Appl. Cryst.* **45**, 849–854.

[bb6] Frasinyuk, M. S., Bondarenko, S. P. & Khilya, V. P. (2012). *Chem. Heterocycl. Compd*, **48**, 422–426.

[bb7] Fun, H.-K., Jebas, S. R., Parveen, M., Khanam, Z. & Ghalib, R. M. (2009). *Acta Cryst.* E**65**, o1322–o1323. 10.1107/S1600536809018029 PMC2969715 21583178

[bb8] Groom, C. R. & Allen, F. H. (2014). *Angew. Chem. Int. Ed. Engl.* **53**, 662–671. 10.1002/anie.201306438 24382699

[bb9] Hussain, H., Hussain, J., Al-Harrasi, A. & Krohn, K. (2012). *Tetrahedron*, **68**, 2553–2578.

[bb10] Ilyas, M. & Parveen, M. (1996). *Tetrahedron*, **52**, 3991–3996.

[bb11] Nardelli, M. (1996). *J. Appl. Cryst.* **29**, 296–300.

[bb12] Panichayupakaranant, P., Noguchi, H. & De-Eknamkul, W. (1998). *Planta Med.* **64**, 774–775. 10.1055/s-2006-957583 17253327

[bb13] Pereira Silva, P. S., Parveen, M., Ali, A., Malla, A. M. & Ramos Silva, M. (2011). *Acta Cryst.* E**67**, o201. 10.1107/S160053681005244X PMC3050332 21522703

[bb14] Sheldrick, G. M. (1996). *SADABS*. University of Göttingen, Germany.

[bb15] Sheldrick, G. M. (2008). *Acta Cryst.* A**64**, 112–122. 10.1107/S0108767307043930 18156677

[bb16] Spek, A. L. (2009). *Acta Cryst.* D**65**, 148–155. 10.1107/S090744490804362X PMC2631630 19171970

[bb17] Tepaske, M. R. & Gloer, J. B. (1992). *J. Nat. Prod.* **55**, 1080–1086.

[bb18] Ulubelen, A., Terem, B. & Tuzlaci, E. (1986). *J. Nat. Prod.* **49**, 692–694. 10.1021/np50046a026 3783166

[bb19] Watkin, D. J., Prout, C. K. & Pearce, L. J. (1996). *CAMERON*. Chemical Crystallography Laboratory, University of Oxford, England.

[bb20] Zhan, Q. F., Xia, Z. H., Wang, J. I. & Lao, A. N. (2003). *J. Asian Nat. Prod. Res.* **5**, 303–306. 10.1080/1028602031000111978 14604241

[bb21] Zhou, H. Y., Hong, J. L., Shu, P., Ni, Y. J. & Qin, M. J. (2009). *Fitoterapia*, **80**, 283–285. 10.1016/j.fitote.2009.03.005 19306914

